# The Determinants of Choosing Traditional Korean Medicine or Conventional Medicine: Findings from the Korea Health Panel

**DOI:** 10.1155/2015/147408

**Published:** 2015-06-25

**Authors:** Ji Heon Choi, Sungwook Kang, Chang Hoon You, Young Dae Kwon

**Affiliations:** ^1^International Institute of Health, No. 402, 30 Digital-ro 32-gil, Guro-gu, Seoul 152-777, Republic of Korea; ^2^Department of Public Health, Daegu Haany University, 1 Haanydaero, Gyeongsan-si, Gyeongsangbuk-do 712-715, Republic of Korea; ^3^Department of Humanities and Social Medicine, College of Medicine and Catholic Institute for Healthcare Management, The Catholic University of Korea, 222 Banpo-daero, Seocho-gu, Seoul 137-701, Republic of Korea

## Abstract

The purpose of this study is to identify key factors that determine a person's decision to seek treatment from traditional Korean medicine (TKM) instead of conventional medicine through analysis of nationally representative data from Korea, where a dual healthcare system exists. The analysis is based on episodic data from the 2008 and 2009 Korea Health Panel. The main dependent variable is the selection between TKM and conventional medicine. We used a multiple logistic regression model to identify the determinants of TKM while controlling for clustered error. Approximately 5% of all doctor's visits were characterized as TKM services. Urban residents were 1.441 times more likely to use TKM than rural residents (*P* = 0.001). The probability of choosing TKM over conventional medicine for a range of conditions compared to the reference condition (gastrointestinal disease) was as follows: circulatory system diseases (OR 5.267, *P* < 0.001), nervous system diseases (OR 12.054, *P* < 0.001), musculoskeletal system diseases (OR 20.579, *P* < 0.001), and neoplasms (OR 0.209, *P* = 0.004). Certain diseases are significantly more likely to be treated by TKM than by conventional medicine. This suggests that many people view TKM as being additionally effective for specific diseases, particularly musculoskeletal disorders.

## 1. Introduction

Recently, in Korea and around the world, interest and research in complementary and alternative medicine (CAM) have been increasing [1–3]. CAM in Western countries incorporates traditional medicines from eastern countries, such as Korea and China, to both complement and compensate for the limitations of conventional medicine [4–7].

In Korea, traditional Korean Medicine (TKM) was widely used before the introduction of conventional medicine at the end of the nineteenth century [8–10]. The efficacy of TKM has been validated by many studies [11–16]. Even after conventional medicine was introduced and established as the standard treatment in South Korea, TKM continued to be officially recognized by the government and has been integrated into a dual healthcare system along with conventional medicine to create a system that is completely unique to South Korea. The Korean dual healthcare system maintains separate physician licenses, educational institutions, and medical facilities for provision of TKM or conventional medicine. Under the dual healthcare system, patients can freely use TKM or conventional medicine based on their choice. Furthermore, cooperative and referral service between TKM and conventional medicine is not common, and the selection of a medical service provider is solely based on the need and judgment of the patient.

TKM has been covered by Korea's mandatory National Health Insurance (NHI) since 1987, which came into effect 10 years after coverage for conventional medicine was put in place [10]. In 1987, NHI reimbursement coverage for TKM included physician consultation fees, acupuncture, moxibustion, cupping, and herbal medication and has gradually expanded to encompass some TKM physical therapies since 2009 [17]. Nevertheless, TKM use in Korea only accounts for a small proportion of total use in healthcare services, and public interest in TKM has been declining. According to the NHI claims database, TKM only accounts for 5.0% of the total use in health care services. Moreover, a survey regarding TKM use reported that the rate of prior experience with TKM had decreased from 86.0% in 2008 to 77.5% in 2011; treatment satisfaction declined from 73.8% (2008) to 56.2% (2011); and public trust in TKM decreased from 72.0% (2008) to 44.9% (2011) [18]. Although the Korean government has implemented various policies and established TKM-related departments and research centers to revitalize utilization of traditional medicine, there is a consensus among TKM providers that traditional medicine has been in crisis since the mid-2000s [19, 20].

Some studies have identified characteristics or preferences of TKM users and determinants of TKM use [21–30]. However, previous studies have produced inconsistent results depending on study samples, analysis methods, and so forth. Most of the research did not utilize nationally representative data, and studies using nationally representative data were not appropriate for a medical utilization study because the data did not focus on medical utilization. Additionally, some of the TKM studies reported only univariable analysis without controlling for confounding factors. Generally, medical utilization is closely related to a patient's illness. It is difficult for patient-based analysis to control for the disease and expenditure of a patient's visit to a physician because a majority of the utilization data is not able to supply the patient's information from a doctor's visit. Therefore, episode-based analysis is more reasonable than patient-based analysis to study the determinants of medical utilization [31].

The purpose of this study is to identify the key factors that determine the use of TKM by analyzing the Korea Health Panel (KHP) which focused on both data collection for medical utilization and nationally representative data from Korea, in which a dual healthcare system exists.

## 2. Methods

The analyses from this research are based on data from the KHP that were collected in 2008 and 2009. The KHP is a nationally representative sample of Korean individuals and their families that include data on demographic and socioeconomic characteristics, health status, access to health care, and private health insurance status. The KHP uses a stratified multistage probability sampling design according to region and residence in order to select nationwide subjects from the 2005 Korea Census. When analyzing these data, we applied cross-sectional weights to account for the multistage sampling design. From the 7,866 households (24,616 individuals) that participated in face-to-face interviews in 2008, 91.5% were recontacted to be interviewed again in 2009.

KHP data has several advantages. For instance, KHP provides information regarding medical utilization in the past year. In addition, KHP provides episode-based data which includes information on physician visits, diagnosis of disease, treatment used, medical cost, and type of medical institution. Another advantage of the KHP, for our purposes, is that the utilization of TKM, as well as conventional medicine, was investigated as part of the survey. What is more, the KHP includes data on health conditions and health behaviors that affect the likelihood of using medical services. It also contains data on physician visits that allow us to determine whether the treatment used traditional or conventional services, and this information was used to explore the determinants for seeking TKM.

This study was approved by the Institutional Review Board of the Catholic University of Korea with a waiver for informed consent because the data were obtained from a public database (https://www.khp.re.kr:444/).

The KHP identifies three types of medical services: inpatient services, physician visits, and emergency services. We focus on physician visits because TKM services are mainly provided in clinics or other outpatient settings. This study examines TKM services, such as acupuncture, moxibustion, and so forth. The use of TKM or conventional medicine is the main dependent variable. We defined “TKM services” as having a physician visit to treat a specific condition in a TKM clinic or hospital. Also, conventional medicine is defined as undergoing a physician visit for treatment in a conventional clinic or hospital.

We principally analyzed three types of independent variables: demographic characteristics, socioeconomic status (SES), and health-related variables. The demographic characteristics included age, sex, and marital status. For SES, we used the logarithm of total household assets, which consisted of the sum of all real estate and financial assets of the household. The residence variable was coded as urban or rural and was intended to serve as a proxy for measuring access to medical services. Binary variables were used for health insurance status.

We analyzed two health-related variables such as disability and chronic disease. We also recorded the number of chronic diseases (e.g., hypertension, diabetes, and cancer) each patient suffered from. Chronic diseases were only considered in this measure if diagnosed by a doctor. Variables reflecting smoking and drinking habits were generated from a supplementary survey. Regular exercise behaviors refer to whether or not the respondents spent at least half an hour on moderate or vigorous physical activity at least three times per week. Lastly, we included the respondents' unmet medical needs as to whether there was a time that they needed health care but did not receive it, or whether they had to forgo health care in the previous year.

We used simple frequencies to describe the characteristics of the episodes based on the type of medical services a patient received, and we used chi-square and ANOVA tests to determine the effects of sociodemographic characteristics and health-related variables on the type of medical treatment sought.

Logistic regression models were used to examine the determinants of choosing TKM through analysis of episodic data while controlling for clustered error. Some individuals in the survey had more than one physician visit, and these episodes violate the statistical assumption of independence. In the presence of clustered error, ordinary linear regression estimates or logistic regression estimates were unbiased, but the standard error may be incorrect. In order to solve issues related to clustered error, regression models that control for this type of error are highly recommended [32]. We present the odds ratios and the 95% confidence intervals, simple frequencies, the results of chi-square and ANOVA tests, and estimates from logistic regression for the determinants of TKM choice. We also present the analysis results that were uncontrolled for clustered error (see Appendix: Table 3). All statistical tests were conducted using STATA 9.1 (StataCorp, College Station, TX, USA).

## 3. Results

### 3.1. Descriptive Statistics per Episode

Approximately 5% of all physician visits were for TKM services during 2008 and 2009. Our study reveals significant differences across all variables between the conventional medicine group and the TKM group. TKM users had a greater ratio of females and people who live in urban areas. On the other hand, there were a higher number of married and educated people among conventional medicine users. TKM users had a higher percentage of NHI beneficiaries and patients using private health insurance, and moreover the household income level of TKM users was higher than that of conventional medicine users. TKM users scored lower on subjective health status and higher on the number of chronic diseases, which represents an objective measure of health status. The TKM and conventional medicine users were clinically similar in health status, even though there was a statistically significant difference between them. Seventy percent of the TKM group was treated in an outpatient clinic for their musculoskeletal problems, while only 26.6% of the conventional medicine group had outpatient treatment for similar complaints. Regarding health behaviors, TKM users were more likely to be nonsmokers and nondrinkers and to be less likely to exercise regularly, compared to the conventional medicine group. Finally, unmet needs for medical services were higher in the TKM group than in the conventional medicine group (Table 1).

### 3.2. Determinants of TKM Utilization

Table 2 shows estimates obtained from a logistic regression that examined the determinants of TKM usage versus conventional medicine usage. Area of residence and presence of a disease that required outpatient treatment were statistically significant predictors of TKM use; no other variables had a significant association with the type of medicine sought. Urban residents were 1.441 times more likely to use TKM than rural residents (*P* = 0.001). The probability of choosing TKM over conventional medicine for a range of conditions compared to the reference condition (gastrointestinal disease) was as follows: circulatory system diseases (OR 5.267, *P* < 0.001), nervous system diseases (OR 12.054, *P* < 0.001), musculoskeletal system diseases (OR 20.579, *P* < 0.001), and neoplasms (OR 0.209, *P* = 0.004). Patients with circulatory, nervous, and particularly musculoskeletal diseases made more visits to TKM institutions than patients with other categories of disease. Cancer patients were least likely to use traditional medicine (Table 2).

## 4. Discussion

The objective of this study was to distinguish the determinants of patient choice of TKM over conventional medicine within the context of a dual health care system that exists in Korea. This study found that the type of disease and the area of residence were key factors for choosing TKM. The reason for a higher rate of TKM use among people in urban areas compared to those in rural areas could be due to easier access to TKM in urban areas. Although the area of residence has an impact on TKM use, the most significant determinant was the patient's illness (i.e., musculoskeletal system diseases: OR 20.579, nervous system diseases: OR 12.054). The high proportion of TKM use among patients with neurological conditions, second only to that of musculoskeletal patients, could be attributed to people seeking treatment after a stroke.

There were obvious differences in TKM use across the different disease types. People generally preferred TKM for problems related to the nervous system (OR 12.054) and the musculoskeletal system (OR 20.579). According to a nationwide study of TKM use conducted in 2011, approximately 70.0% of all TKM users were musculoskeletal patients [30]. Moreover, NHI claim data from 2009 show that more than 50.0% of patients visited centers for TKM to treat their musculoskeletal problems, suggesting that TKM visits mainly focus on treating this specific disease group. The high proportion of TKM use among patients with neurological conditions, second only to that of musculoskeletal patients, could be due to people seeking treatment after a stroke. Stroke, which is the second highest cause of mortality in South Korea, is often treated with TKM for rehabilitation purposes [33]. We found that cancer patients are the least likely to use TKM, which we attribute to TKM not being used as a standard treatment for cancer but used only as either an adjuvant therapy or in a limited capacity for terminal stage patients [34, 35].

The higher rate of TKM use among people from urban areas compared to those in rural areas could be attributed to easier access to TKM in urban areas. According to statistics from the Health Insurance Review and Assessment Service (2011), the number of centers for TKM is 25.4 per 100,000 people living in urban areas compared to 18.7 per 100,000 people living in rural areas [18]. In a previous study, regional medical utilization between TKM and conventional medicine was not significantly different [36]. However, a recently conducted study of elderly people reported that the frequency of TKM use is considerably higher among urban residents than among rural residents [27], which is consistent with the findings of this study. Although the area of residence has an important influence on TKM use, the most significant determinant was the type of disease. This study did not find significant demographic or socioeconomic factors except for the area of residence. These results imply that TKM providers have focused on specific diseases and have failed to extend their services beyond them. It is also possible that TKM users consider TKM to be limited in its ability to treat other diseases and are beginning to question its effectiveness.

The results of this study show that the area of residence and the type of disease have the biggest influence on a person's choice to utilize TKM. Apart from showing that TKM use is more common among females than among males [26, 28], we found no significant gender differences in the utilization of TKM. Furthermore, our findings are inconsistent with previous studies that report a significant association between TKM use and increasing age [26]; although we found age differences in TKM use (30–39-year-olds and 40–49-year-olds used TKM more than 20–29-year-olds, and 20–29-year-olds used TKM more frequently than adults ≥ 50 years), they were not significant after controlling for clustered errors. Previous studies analyzing CAM users found that women, middle-aged adults, and people with higher educational backgrounds were more likely to use CAM [2, 37–40]. Though our results show the same pattern, they are not significant after controlling for clustered error.

To date, most studies regarding TKM use have been conducted by examining traditional medicine users and focused on their individual characteristics [25–29]. Even though there have been several national studies that compared TKM users with non-TKM users through analysis of a large sample from the South Korean population, the majority of this research only focused on comparing TKM users with non-TKM users rather than looking into the factors that determine the choice to seek TKM treatment instead of conventional treatment [28, 29]. Because TKM users may also receive conventional medical treatment, it is more appropriate to identify the determinants of TKM use as opposed to conventional medical treatment for specific health concerns. And since South Korea maintains a dual health care system, in which both TKM and conventional medicine coexist, it is important to identify factors that predict the use of TKM over conventional medicine.

Most of the people this study analyzed either used only conventional medicine or used both conventional medicine and TKM within Korea's dual healthcare system. We divided medical utilization into two categories: episode of TKM use and episode of conventional medicine use. Subsequently, we performed a logistic regression model to examine the determinants of choice of TKM use compared to conventional medicine by using episodic data. Previous studies that analyzed the use of health care services by individual characteristics examined each episode, but this approach could potentially decrease the reliability of the regression model because it ignored unobserved individual characteristics. In the present study, we analyzed the data per episode but we also controlled for unobserved characteristics by clustering individual characteristics. When clustered errors were not controlled for, we observed results similar to previous studies; age, level of education, types of health insurance, income level, health status, and health behaviors were significant factors affecting the use of TKM. However, when we controlled for the clustered errors, we found that only the area of residence and the disease type were significant factors of choosing TKM.

We conclude that our results indicate that an interest in TKM is still low even though the rate of TKM use remains unchanged. The proportion of TKM services remains constant since the 1990s and is estimated to be approximately 5% of the entire health care industry [18]. Despite government efforts to expand TKM services through greater NHI coverage and the fact that the dual health care system enables patients to easily access conventional and traditional medicine, we found a strong preference among the public to seek TKM treatment only for specific conditions.

According to the results of the 2011 survey, half of the TKM users were unsatisfied with their treatment because they believed that the treatment was ineffective [18]. Also, the overall dissatisfaction rate of TKM increased from 2008 to 2011. Survey respondents reported that the safety of herbal medicines, the high cost physician visits, the unreliability of the treatment's effectiveness, and the professionalism of TKM needed to be improved [18]. On the other hand, it has been reported that TKM providers support efforts to obtain clinical results and develop therapeutic methodologies so that TKM can become scientifically established as an evidence-based practice [18, 41].

This study has a number of limitations. First of all, we could not identify the chronological order of TKM use and conventional medicine use in the database for patients with more than one doctor's visit. Further studies tracking patients' use of healthcare services should allow for analysis of the chronological order of visitations to determine whether conventional medicine and TKM are sought in conjunction or as substitutes for each other. Secondly, this study is not generalizable beyond Korea because there are different cultural contexts in other countries as well as different reimbursement systems. Study results can be misleading when some facts are not taken into consideration with regards to the health care delivery system and health-related policies and regulations that affect the use of TKM. Thirdly, in order to control for estimates using univariate test such as trend and chi-square test, some results have a possibility of inherent statistical inaccuracies because of small frequency in some disease categories.

## 5. Conclusions

Overall, we showed that some categories of diseases are more likely to be treated by TKM than conventional medicine. This indicates that people view TKM as being additionally effective for specific diseases, particularly musculoskeletal disorders. However, we found that a majority of people still were not heavily interested in TKM. Evidence-based therapeutic methodologies and treatment outcomes for TKM should be established and refined to change the public perception of TKM and improve public trust in TKM.

## Figures and Tables

**Table 1:** Descriptive statistics on distribution of physician visit per episode^*∗*^ (*n* = 191,449).

	Conventional medicine		Traditional Korean medicine		Total		*P* value
	*n*	%	*n*	%	*n*	%	
Sex							<0.0001
Female	115,581	63.5	6,587	69.4	122,168	63.8	
Male	66,379	36.5	2,902	30.6	69,281	36.2	
Age (years)							<0.0001
20–29	8,009	4.4	332	3.5	8,341	4.4	
30–39	17,212	9.5	878	9.3	18,090	9.5	
40–49	24,345	13.4	1,522	16.0	25,867	13.5	
50–59	34,651	19.0	1,692	17.8	36,343	19.0	
60–69	46,856	25.8	2,321	24.5	49,177	25.7	
≥70	50,887	28.0	2,744	28.9	53,631	28.0	
Marital status							<0.0001
Married	132,704	73.2	6,599	69.6	139,303	73.0	
Divorced	38,649	21.3	2,392	25.2	41,041	21.5	
Single	10,047	5.5	484	5.1	10,531	5.5	
Education level							<0.0001
Elementary school	74,708	41.1	4,260	44.9	78,968	41.3	
Middle school	27,655	15.2	1,231	13.0	28,886	15.1	
High school	47,209	25.9	2,345	24.7	49,554	25.9	
College or higher	32,388	17.8	1,653	17.4	34,041	17.8	
Residence							<0.0001
Rural	117,205	64.4	5,707	60.1	122,912	64.2	
Urban	64,755	35.6	3,782	39.9	68,537	35.8	
Health insurance type							0.0115
Medical Aid	17,752	9.8	851	9.0	18,603	9.7	
National Health Insurance	164,208	90.2	8,638	91.0	172,846	90.3	
Private health insurance							0.028
No	87,589	48.1	4,458	47.0	92,047	48.1	
Yes	94,371	51.9	5,031	53.0	99,402	51.9	
Household income (dollars)	23,103 ± 21,169		24,012 ± 20,157		23,198 ± 21,067		<0.0001
Subjective health status (100 pts)	65.4 ± 18.1		64.9 ± 18.6		65.4 ± 18.7		0.0057
Number of chronic diseases	3.05 ± 2.43		3.20 ± 2.52		3.06 ± 2.44		0.0004
Disease							<0.0001
Neoplasm	2,876	1.6	10	0.1	2,886	1.6	
Endocrine system	8,337	4.7	21	0.2	8,358	4.5	
Nervous system	3,099	1.7	71	0.8	3,170	1.7	
Eye and ear	6,556	3.7	27	0.3	6,583	3.5	
Circulatory system	25,802	14.6	937	10.3	26,739	14.4	
Respiratory system	25,996	14.7	138	1.5	26,134	14.0	
Dental disease	11,844	6.7	0	0.0	11,844	6.4	
Gastrointestinal disease	9,265	5.2	160	1.8	9,425	5.1	
Skin and subcutaneous tissue	6,239	3.5	100	1.1	6,339	3.4	
Musculoskeletal system	47,166	26.6	6,350	70.0	53,516	28.7	
Genitourinary system	7,446	4.2	93	1.0	7,539	4.1	
Others	22,657	12.8	1,163	12.8	23,820	12.8	
Smoking							<0.0001
Current smoker	59,757	33.5	2,755	29.4	62,512	33.3	
Past smoker	1,590	0.9	105	1.1	1,695	0.9	
No smoking history	117,244	65.6	6,508	69.5	123,752	65.8	
Drinking							<0.0001
No	55,328	31.0	2,622	28.0	57,950	30.8	
Yes	123,263	69.0	6,746	72.0	130,009	69.2	
Regular exercise							<0.0001
No	135,949	76.1	7,333	78.3	143,282	76.2	
Yes	42,642	23.9	2,035	21.7	44,677	23.8	
Unmet need							0.0001
No	145,043	81.2	7,458	79.6	152,501	81.1	
Yes	33,548	18.8	1,910	20.4	35,458	18.9	

^*∗*^Clustered error correction in univariable analysis.

**Table 2:** Determinants of choosing traditional Korean medicine after adjusting for clustered error.

	Odds ratio	95% CI	*P* value
Sex (ref = female)	1.041	0.713–1.158	0.834
Age (ref = 20–29)			
30–39	1.236	0.726–2.104	0.435
40–49	1.322	0.744–2.351	0.341
50–59	0.866	0.476–1.572	0.637
60–69	0.828	0.449–1.525	0.545
≥70	0.734	0.373–1.442	0.370
Marital status (ref = single)			
Married	0.978	0.589–1.624	0.934
Divorced	1.152	0.633–2.096	0.642
Education (ref = elementary school)			
Middle school	0.903	0.666–1.225	0.514
High school	1.022	0.763–1.367	0.883
College or higher	1.089	0.739–1.606	0.665
Residence (ref = rural)	1.441	1.155–1.797	0.001
Health insurance type (ref = MA)	1.384	0.846–2.261	0.195
Private health insurance (ref = no)	1.061	0.856–1.315	0.588
Household income (log)	0.972	0.933–1.013	0.187
Subjective health status	1.002	0.996–1.008	0.458
Number of chronic diseases	1.018	0.968–1.072	0.476
Disease (ref = gastrointestinal)			
Neoplasm	0.209	0.071–0610	0.004
Endocrine system	0.370	0.119–1.150	0.086
Nervous system	12.054	6.142–23.658	<0.001
Eye and ear	0.774	0.295–1.876	0.531
Circulatory system	5.267	2.726–10.178	<0.001
Respiratory system	0.604	0.270–1.352	0.220
Skin and subcutaneous tissue	1.006	0.408–2.481	0.989
Musculoskeletal system	20.579	12.353–34.282	<0.001
Genitourinary system	1.678	0.734–3.834	0.219
Others	6.294	3.717–10.675	<0.001
Smoking (ref = no smoking history)			
Current smoker	0.972	0.695–1.361	0.872
Past smoker	1.318	0.691–2.512	0.401
Drinking (ref = no)	0.993	0.798–1.236	0.956
Regular exercise (ref = no)	0.853	0.683–1.064	0.159
Unmet need (ref = no)	1.070	0.861–1.331	0.538

Number of observations	179,026		
−2 log likelihood	−30,715.41		
Wald test	557.38 (*P* < 0.0001)		
Pseudo *R* ^2^	0.1373		

ref: reference; MA: Medical Aid; CI: confidence interval.

**Table 3:** Determinants of choosing traditional Korean medicine without adjusting for clustered error.

	Odd Ratios	95% CI	*P* value
Sex (ref = female)	1.041	0.965–1.122	0.291
Age (ref = 20–29)			
30–39	1.236	1.036–1.473	0.018
40–49	1.322	1.108–1.578	0.002
50–59	0.866	0.719–1.042	0.129
60–69	0.828	0.684–1.002	0.053
≥70	0.734	0.602–0.895	0.002
Marital status			
Married	0.978	0.850–1.126	0.767
Divorced	1.152	0.989–1.341	0.067
Education (ref = elementary school)			
Middle school	0.903	0.840–0.970	0.006
High school	1.022	0.954–1.094	0.883
College or higher	1.089	1.000–1.187	0.050
Residence (ref = rural)	1.441	1.375–1.509	<0.001
Health insurance type (ref = MA)	1.384	1.273–1.503	<0.001
Private health insurance (ref = no)	1.061	1.003–1.121	0.036
Household income (log)	0.972	0.961–0.983	<0.001
Subjective health status	1.002	1.001–1.003	0.001
Number of chronic diseases	1.018	1.007–1.029	0.001
Disease (ref = gastrointestinal)			
Neoplasm	0.209	0.077–0.566	0.002
Endocrine system	0.370	0.234–0.584	<0.001
Nervous system	12.054	10.003–14.526	<0.001
Eye and ear	0.774	0.533–1.038	0.082
Circulatory system	5.267	4.429–6.624	<0.001
Respiratory system	0.604	0.270–1.352	0.220
Skin and subcutaneous tissue	1.006	0.725–1.397	0.969
Musculoskeletal system	20.579	17.509–24.187	<0.001
Genitourinary system	1.678	1.295–2.174	<0.001
Others	6.294	5.312–7.459	<0.001
Smoking (ref = no smoking history)			
Current smoker	0.972	0.904–1.045	0.454
Past smoker	1.318	1.065–1.630	0.011
Drinking (ref = no)	0.993	0.955–1.033	0.758
Regular exercise (ref = no)	0.853	0.807–0.901	<0.001
Unmet need (ref = no)	1.070	1.014–1.130	0.014

Number of observations	179,026		
−2 log likelihood	−30,715.41		
LR test	9,775.68 (*P* < 0.0001)		
Pseudo *R* ^2^	0.1373		

ref: reference; MA: Medical Aid; CI: confidence interval.

## Appendix

See Table 3.
